# Cardio-Metabolic Effects of HIV Protease Inhibitors (Lopinavir/Ritonavir)

**DOI:** 10.1371/journal.pone.0073347

**Published:** 2013-09-30

**Authors:** Kathleen M. S. E. Reyskens, Tarryn-Lee Fisher, Jonathan C. Schisler, Wendi G. O'Connor, Arlin B. Rogers, Monte S. Willis, Cynthia Planesse, Florence Boyer, Philippe Rondeau, Emmanuel Bourdon, M. Faadiel Essop

**Affiliations:** 1 Cardio-Metabolic Research Group (CMRG), Department of Physiological Sciences, Stellenbosch University, Stellenbosch 7600, South Africa; 2 McAllister Heart Institute, Department of Pathology & Laboratory Medicine, University of North Carolina, Chapel Hill, North Carolina, USA; 3 Groupe d'Etude sur l'Inflammation Chronique et l'Obésité (GEICO), Plateforme CYROI, Université de La Réunion, Saint Denis de La Réunion, France; Texas A & M, Division of Cardiology, United States of America

## Abstract

Although antiretroviral treatment decreases HIV-AIDS morbidity/mortality, long-term side effects may include the onset of insulin resistance and cardiovascular diseases. However, the underlying molecular mechanisms responsible for highly active antiretroviral therapy (HAART)-induced cardio-metabolic effects are poorly understood. In light of this, we hypothesized that HIV protease inhibitor (PI) treatment (Lopinavir/Ritonavir) elevates myocardial oxidative stress and concomitantly inhibits the ubiquitin proteasome system (UPS), thereby attenuating cardiac function. Lopinavir/Ritonavir was dissolved in 1% ethanol (vehicle) and injected into mini-osmotic pumps that were surgically implanted into Wistar rats for 8 weeks vs. vehicle and sham controls. We subsequently evaluated metabolic parameters, gene/protein markers and heart function (*ex vivo* Langendorff perfusions). PI-treated rats exhibited increased serum LDL-cholesterol, higher tissue triglycerides (heart, liver), but no evidence of insulin resistance. In parallel, there was upregulation of hepatic gene expression, i.e. acetyl-CoA carboxylase β and 3-hydroxy-3-methylglutaryl-CoA-reductase, key regulators of fatty acid oxidation and cholesterol synthesis, respectively. PI-treated hearts displayed impaired cardiac contractile function together with attenuated UPS activity. However, there was no significant remodeling of hearts exposed to PIs, i.e. lack of ultrastructural changes, fibrosis, cardiac hypertrophic response, and oxidative stress. Western blot analysis of PI-treated hearts revealed that perturbed calcium handling may contribute to the PI-mediated contractile dysfunction. Here chronic PI administration led to elevated myocardial calcineurin, nuclear factor of activated T-cells 3 (NFAT3), connexin 43, and phosphorylated phospholamban, together with decreased calmodulin expression levels. This study demonstrates that early changes triggered by PI treatment include increased serum LDL-cholesterol levels together with attenuated cardiac function. Furthermore, PI exposure inhibits the myocardial UPS and leads to elevated calcineurin and connexin 43 expression that may be associated with the future onset of cardiac contractile dysfunction.

## Introduction

The human immunodeficiency virus (HIV) has infected over 40 million individuals over the last decade, with more than 5 million residing in sub-Saharan Africa [1], [2]. Although highly active antiretroviral therapy (HAART) enhances life expectancy and quality of infected individuals [3], [4], there is increased emphasis on HAART-mediated metabolic derangements [5] and its potential risk for cardiovascular diseases (CVD) in the long-term.

Protease inhibitors (PIs) form an integral part of HAART and side-effects include development of dyslipidemia, i.e. greater production of plasma triglycerides and lipids together with an adverse cholesterol profile [6]–[8]. Together such derangements elicit inflammation, stress the myocardium (9), and may potentially predict the onset of insulin resistance (IR) [10], [11] and cardiac dysfunction (11). PIs are also linked to increased risk for myocardial infarction [13] and cardiovascular abnormalities [14], [15], with many changes resembling coronary artery disease [16]. It is unclear whether metabolic side effects of PIs are independently and/or causally linked with cardiovascular perturbations. Moreover, the effects of PIs *per se* on the heart in this context are also poorly understood. Therefore, an emerging focus is to identify key metabolic and transcriptional pathways that may mediate PI-induced cardio-metabolic pathophysiology. For example, we recently found that rats exposed to 8 weeks of PI treatment displayed cardiac dysfunction [17]. Moreover, PI-treated HIV-infected individuals exhibit elevated reactive oxygen species (ROS) production [18]–[20] that may trigger the activation of detrimental signaling and cell death pathways [21].

HIV-PIs may also exert unfavorable effects at the gene transcriptional level, e.g. activating sterol regulatory element binding protein (SREBP) [22], a key lipid transcriptional modulator expressed in major metabolic tissues [23]. Upon activation, SREBP binds to sterol-regulatory-element (SRE)-containing promoter sequences in lipogenic and cholesterogenic genes (e.g. 3-hydroxy-3-methyl-glutaryl-CoA reductase [*hmgcr*]) that ultimately results in the production of cholesterol (high and low density lipoproteins) and sterol components [24]. The ubiquitin-proteasome system (UPS) – responsible for removal of misfolded or damaged proteins – is also implicated in the onset of such metabolic side effects. For example, the PI Ritonavir attenuates chymotrypsin- and trypsin-like activities of the 20S UPS subunit in hepatocytes [25]. As a result, degradation of apolipoprotein B (major determinant of plasma lipid levels) was diminished thus providing a potential mechanism for PI-induced hyperlipidemia [26]. Furthermore, SREBPs are ubiquitinated and degraded by the UPS [27], [28] raising the possibility that an inhibition of this system may also contribute to development of dyslipidemia in HIV-infected individuals treated with PIs. Together this may establish a pro-atherogenic profile and increase the risk for the onset of CVD.

Despite such progress, the underlying molecular mechanisms responsible for HAART-induced cardio-metabolic side effects are poorly understood, and little is known about the earliest events driving this process. Whether these molecular alterations occur as a direct result of PI treatment or through the activation of additional pathways throughout the body at a later stage remain elusive. For the current study, we therefore hypothesized that PI treatment enhances myocardial oxidative stress and concomitantly inhibits the UPS, having a knock-on effect on important downstream regulators such as gap junctions and ion channels essential in cardiac physiology. We also evaluated several non-oxidative glucose metabolic circuits i.e. the polyol pathway, hexosamine biosynthetic pathway (HBP), advanced glycation end products (AGE), and PKC activation since previous work found its activation can elicit the onset of cardio-metabolic complications [29]. Since our previous *ex vivo* rat heart study [17] implicated altered calcium homeostasis in PI-mediated cardiac dysfunction, we further investigated calcium signaling and mitochondrial energetic regulators in an established rat model of chronic PI drug delivery. These data may explain and suggest an association between molecular changes and depressed cardiac contractile function.

## Materials and Methods

### 

#### Animal model

Lopinavir/Ritonavir (Kaletra^TM^, Abbott Laboratories, Abbot Park IL) was crushed and dissolved in a 1% ethanol (vehicle) solution at human steady-state plasma concentration (7.1±2.9 µg/mL), sterile filtered and injected into a mini-osmotic pump (Alzet, Cupertino CA). Male Wistar rats (180–220 g) received either: mock surgery (sham), vehicle-, or PI-containing pump for a total of 8 weeks (n = 8 per group) as previously described [17]. Food consumption was measured via weekly weighing of the food (in cages) and expressed as average food consumed per rat. All animals were treated in accordance with the Guide for the Care and Use of Laboratory Animals of the National Academy of Sciences (NIH publication No. 85–23, revised 1996) and performed with the approval of the Animal Ethics Committee of Stellenbosch University (South Africa).

#### Baseline heart function assessment

After 8 weeks rats were euthanized with pentobarbitone-sodium (10 mg/kg, i.p.) and hearts rapidly excised, weighed and placed into ice-cold Krebs-Henseleit (KH) buffer before cannulation on a Langendorff perfusion rig as previously described [17]. The cannulation and perfusion occurred within <1.5 min of excision for all hearts. Additional parameters to the ones we have previously published include ±dP/dt and heart rate during 60 min of perfusion.

#### Histologic and metabolic measurements

After 8 weeks, harvested tissues (heart, liver, adipose, pancreas and skeletal muscle) were fixed, processed and embedded in paraffin wax whereafter sections were stained with a) hematoxylin and eosin (H & E) for general morphologic evaluation and b) Sirius red for detection of collagen deposits (fibrosis).

In an identical cohort of animals, we evaluated both serum and tissue metabolite levels following PI treatment. After 8 weeks (4–7 days before termination of treatment period) rats underwent a 12–18 h overnight fast whereafter blood was collected from the jugular vein under anesthesia (isofluorane in oxygen, 5% for induction and 3% for maintenance). Serum was isolated from the collected blood and analyzed for: total and LDL cholesterol, free fatty acids (FFA) and triglyceride (TG) levels (NHLS, Tygerberg Hospital, South Africa). We also evaluated the homeostatic model of assessment for insulin resistance (HOMA-IR) – here serum insulin and glucose levels were also determined (PathCare Laboratory, Stellenbosch, South Africa). The HOMA-IR was calculated as follows: (glucose [mg/dL] x fasting insulin [μU/mL]/2.43) and the equation used in accordance with the guidelines for HOMA-IR assessment in rodents [30].

Isolated heart and liver tissues were also assessed for: total cholesterol, HDL, LDL/VLDL cholesterol (Abcam, Cambridge MA) and TG content (BioVision, Milpitas CA) according to the manufacturer's instructions.

#### Real-time qPCR analysis for gene expression

Total RNA was isolated from homogenized liver and heart tissues (n = 8) using the RNeasy® Mini Kit (Qiagen, Germantown, MD) according to the manufacturer's protocols as previously described [31], [32]. First strand cDNA was made using the iScript™ cDNA synthesis kit (BioRad, Hercules CA) using 200 & 250 ng of RNA from liver, and heart tissue, respectively, and included the Solaris synthetic RNA Spike Control (Thermo Scientific, Waltham MA) to test for reverse transcription and PCR inhibition. Samples that did not show significant inhibition (ΔC_q_ <3 compared to synthetic target alone, 69 of 72 samples) were diluted 20-fold in water and used for gene expression analysis. A total of three samples exhibited inhibition and were not used for qPCR analysis. We evaluated expression of the following genes: acetyl-CoA carboxylase isoforms (*accα*: marker of FA synthesis; *accβ*: marker of FA oxidation); fatty acid synthase (*fasn*: marker of FA synthesis); glycerol-3-phosphate acyltransferase (mitochondrial) (*gpam*: marker of glycero-lipid synthesis); hydroxyl-3-methyl-glutaryl-CoA reductase (*hmgcr*: marker of cholesterol synthesis); LDL receptor (*ldlr*: marker of LDL metabolism); SREBP isoforms (*srebpf1/2*: evaluate role of SREBPs); and glutamine fructose-6-phosphate amidotransferase (*gfat1*: HBP marker). For all qPCR reactions, 2 μL of diluted cDNA (range ∼2–5 ng of cDNA) was used in technical triplicate reactions using LightCycler® 480 Probes Master mix (Roche, Indianapolis IN) for 5′ exonuclease chemistry with primers and probes per manufacturer specifications (Primers [forward/reverse]: *actb –* cccgcgagtacaaccttct/cgtcatccatggcgaact; *hprt1* – gaccggttctgtcatgtcg/acctggttcatcatcactaatcac; *pgk1* – ccagataacgaataaccaaagga/gacttggctccattgtcca; *gapdh* – agctggtcatcaatgggaaa/ atttgatgttagcgggatcg; *g6pdh* – ttatcatcatgggtgcatcg/aaggtgtcttcgggtagaagg; *gusb* – ctctggtggccttacctgat/cagactcaggtgttgtcatcg; *tbp* – cccaccagcagttcagtagc/ cccaccagcagttcagtagc; RNA – tgcaagccaattcccgaag/ ccattgtagtgaacagtaggac and Probes: *18S* – Hs99999907_s1; *acaca* – Rn00573474_m1; *acacb* – Rn00588290_m1; *fasn* – Rn00569117_m1; *gfpt1* – Rn01765492_m1; *gpam* – Rn00568620_m1, *hmgcr* – Rn00565598_m1, *ldlr* – Rn00598442_m1, *srebf1* – Rn01495769_m1; *srebf2* – Rn01502638_m1)(Life Technologies, Grand Island NY; Roche, Indianapolis IN; Thermo Scientific, Waltham MA).

Reactions were run on the LightCycler ® 480 qPCR instrument (Roche, Indianapolis IN). Relative quantification was calculated using the ΔC_q_ method corrected for amplicon efficiencies (range  = 1.9–2.1). Reference gene fitness was determined by measuring a panel of genes: *18s*, *Actb*, *G6pdh*, *Gapdh*, *Hprt1*, *Pgk1*, and *Tbp*. The most stable genes across the three conditions for each tissue was determined using the NormFinder algorithm [33], [34]; subsequently, the GeNorm algorithm [33], [34] was used to calculate the number of reference genes needed to maximize stability. For adipose tissue and liver tissue, three reference genes were utilized (*G6pdh*, *Hprt*, *Pgk1*- variation (V)  = 0.3; and *18s*, *Tbp*, *Gapdh* – V  = 0.1, respectively) and for heart tissue, four reference genes were used (*18s*, *G6pdh*, *Hprt1*, *Tbp* – V = 0.4).

Relative target gene expression levels were determined using the ΔC_q_ method followed by reference gene normalization as described [34].

#### Proteasome activity measurements

Chymotrypsin-like, trypsin-like, and caspase-like activities of the proteasome were assayed using fluorogenic peptides (Sigma-Aldrich, St Louis, MO): Suc-Leu-Leu-Val-Tyr-7-amido-4-methylcoumarin (LLVY-MCA at 25 µM), N-t-Boc-Leu-Ser-Thr-Arg-7-amido-4-methylcoumarin (LSTR-MCA at 40 µM) and N-Cbz-Leu-Leu-Glu-b-naphthylamide (LLE-NA at 150 µM), respectively, as described before by us [35].

#### Western blotting analysis

Total protein was extracted from tissue samples as described [36], while nuclear protein extraction was performed using the high-salt extraction method [37]. Protein concentrations for both total and nuclear lysates were determined by the Lowry method and Western blotting performed for: SREBP-1 for cytosolic (cSREBP-1) and nuclear (nSREBP-1) proteins, calmodulin, calcineurin, phosphorylated calcium/calmodulin-dependent kinase II (pCaMKII), nuclear factor of activated T-cells 3 (NFAT3), phosphorylated phospholamban (pPLB), peroxisome proliferator-activated receptor gamma coactivator-1-alpha (PGC-1α), mitochondrial transcription factor A (mtTFA), nuclear respiratory factor 1 (NRF-1), connexin 43 and ubiquitin. Protein detection was performed using standard methods [36] and expression determined by the adjusted percentage volume (intensity units of band x mm^2^) after background subtraction and normalized to β–actin or Ponceau stain to correct for variations in loading. Primary antibodies (rabbit or mouse) were purchased from Santa Cruz Biotechnologies (Santa Cruz Biotechnologies, Santa Cruz CA) or Cell Signaling (Cell Signaling, Danvers MA) to be used at 1:1000 dilution with corresponding secondary antibody (anti-rabbit or anti-mouse HRP-linked antibodies at 1:4000 dilution).

#### Evaluation of myocardial oxidative stress

Superoxide dismutase (SOD) (Enzo Life Sciences, Farmingdale NY) activity was measured in mitochondrial preparations (prepared according to Boudina *et al*. [38]) following the manufacturer's instructions. The catalase activity assay is based on the properties of catalase enzyme to reduce hydrogen peroxide (H_2_O_2_) into oxygen (O_2_) and water (H_2_O) [39]. Assays were carried on about 80 μg of protein lysate in 25 mM Tris–HCl (pH 7.5). Blanks were measured at 240 nm just before adding 80 µL of H_2_O_2_ (10 mM final) to start the reaction. Catalase activity was determined by measuring the absorbance decrease of H_2_O_2_ at 240 nm. The decomposition of hydrogen peroxide is a first order reaction type following H_2_O_2_ concentration and the rate constant K for the overall reaction is given by:
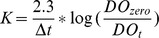



Each measurement was considered with 4 replicates and data are expressed as catalytic unit (U) per mg of total protein. Protein carbonylation was performed on heart tissues as described before [40].

#### Determination of non-oxidative pathway activation

Briefly, collected heart tissues were homogenized with modified ice-cold RIPA buffer, the supernatant centrifuged twice at 13, 000 *g* for 10 min at 4°C then stored at −80°C until further use. We employed Western blotting analysis to determine total *O*-GlcNAc expression as a marker for myocardial HBP activation, and methylglyoxal concentrations to assess AGE pathway activation (OxiSelect™ MG ELISA Kit; Cell Biolabs, San Diego CA). Methylglyoxal derivatives are formed from the non-enzymatic reaction of reducing carbohydrates such as glucose and carbonyl compounds (glyceraldehyde) in the Maillard reaction – products of this reaction are called AGEs. MG levels were calculated from the standard curve and are expressed as nmol per mg protein. The PKC assay was carried out using an ELISA-based method as detailed in the kit's instruction manual (Enzo Life Sciences, Farmingdale NY). PKC activity was determined from the standard curve and expressed as ng/min/mL.

D-sorbitol, an intermediate of the polyol pathway was measured as an index of pathway activation. D-sorbitol levels were measured as detailed in the instructions of a commercially obtained kit (BioVision K 631-100, Mountain View CA). We calculated the sorbitol concentration (C) of samples by using the sample amount (nmol) from the standard curve (S_a_), sample volume (μL) used (S_v_) and the dilution factor (D); C = S_a_/S_v_*D. We used a modified protocol (EC 2.2.1.1) from Sigma Aldrich (St. Louis MO) to determine transketolase activity as a marker of the non-oxidative branch of the pentose phosphate pathway (PPP). Briefly, xylulose 5-phosphate and ribulose 5-phosphate are converted by transketolase in the presence of magnesium ions and thiamine pyrophosphate to glyceraldehyde 3-phosphate and sedoheptulose 7-phosphate. G 3-P is then further converted by triosephosphate isomerase to dihydroxyacetone phosphate, and with the addition of β-NADH and α-glycerophosphate dehydrogenase to produce α-glycerol phosphate and β-NAD to be measured spectrophotometrically at 340 nm. This protocol was adapted from de la Haba *et al.*(1955) [41].

#### Statistical analyses

Data are presented as mean ± SEM, and values considered significant when p<0.05. Statistical analysis was performed by one-way ANOVA, with the exception of heart function (two-way ANOVA). The Bonferroni *post-hoc* test comparing all groups to each other were used to determine differences (GraphPad Prism v5, San Diego CA).

## Results

**Figure 1:**
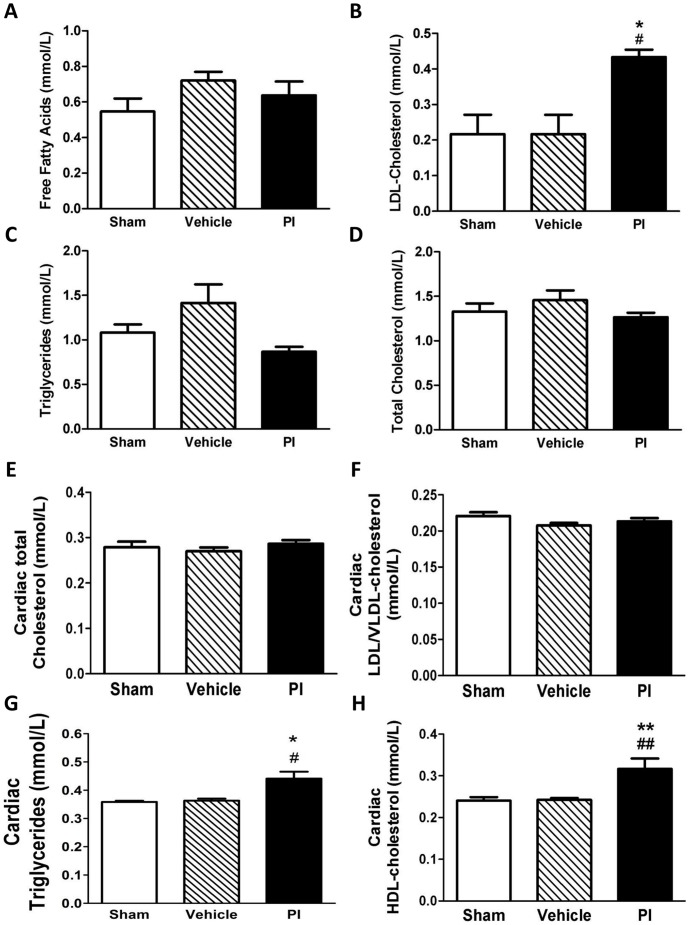
Serum and tissue lipid characterization (n = 8). A) serum FFA; B) serum LDL-cholesterol; C) serum TG; D) serum total cholesterol; E) cardiac total cholesterol; F) cardiac LDL-cholesterol; G) cardiac TG; and H) cardiac HDL-cholesterol. *p<0.05, **p<0.01 vs. sham; #p<0.05, ##p<0.01 vs. vehicle. Data presented as mean ± SEM and one-way ANOVA with Bonferroni *post-hoc* test performed for statistics. *FFA – free fatty acids, HDL – high density lipoprotein, LDL – low density lipoprotein, PI – protease inhibitor, TG – triglyceride*.

Fasting serum levels for FFA, TG, total cholesterol, insulin and glucose were not significantly altered with chronic PI treatment (**Fig.** **1**), with HOMA-IR also not showing any differences (data not shown, p>0.05). However, PI treatment increased serum LDL-cholesterol levels to 0.433±0.021 vs. 0.216±0.005 mM (sham) (p<0.05) and vs. 0.216±0.005 mM (vehicle) (p<0.05). Furthermore, triglyceride content was significantly elevated in heart (**Fig. 1G**) and liver tissues (data not shown), while PI-treated hearts also exhibited higher HDL-cholesterol levels (p<0.01 vs. sham and vehicle) (**Fig. 1H**). No significant elevations were found for cardiac total cholesterol and VLDL-cholesterol content (p>0.05) (**Fig. 1** **E, F**). In addition, total food consumed in PI-treated animals was significantly higher than the control groups; i.e. 894±17.8 g vs. 707±14.3 g (sham) (p<0.05) and vs. 717±13.1 g (vehicle) (p<0.05).

**Figure 2:**
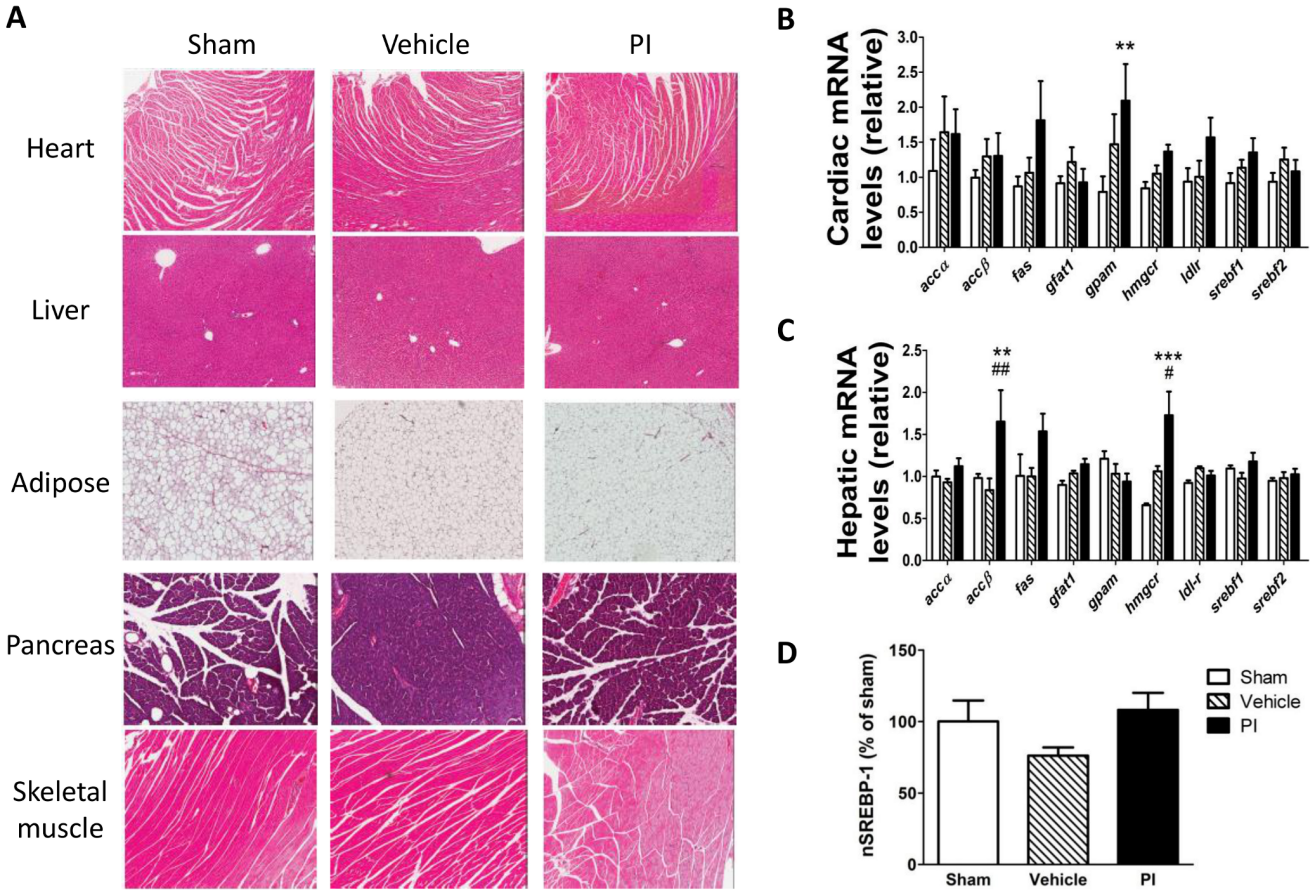
Gene and histologic characterization (n = 8). A) H & E staining for various tissues following PI treatment; B) Heart; and C) liver gene regulation; and D) nuclear SREBP protein expression. **p<0.01, ***p<0.001 vs. sham; #p<0.05, ##p<0.01 vs. vehicle. Data presented as mean ± SEM and one-way ANOVA with Bonferroni *post-hoc* test performed for statistics. *H & E – hematoxylin and eosin, LDL – low density lipoprotein, PI – protease inhibitor, SREBP – sterol regulatory element binding protein, gene abbreviations – refer Methods section.*

Histological analysis (H & E staining) did not reveal major ultrastructural abnormalities for all tissues examined (**Fig. 2A**) while no fibrosis was detected (data not shown). To determine mechanisms responsible for early metabolic changes, we evaluated lipid and cholesterogenic genes in heart and liver tissues. Here PI treatment enhanced cardiac *gpam* expression (p<0.001 vs. sham), although this was not significant vs. vehicle-treated rats (**Fig. 2B**). However, hepatic *accβ* and *hmgcr* expression were significantly upregulated in the PI group (p<0.05 vs. vehicle) (**Fig. 2C**). Since our gene data indicate PI-mediated transcriptional effects, we next assessed the expression of the transcriptional modulator SREBP-1. However, we found no significant alterations with PI treatment for nuclear (**Fig. 2D**) and cytosolic SREBP1 (data not shown).

Further analyses of heart function at baseline revealed significantly decreased contractile force with PI treatment. Here both maximal and minimal contractile forces were altered with 8 weeks of PI therapy (**Table 1**) when compared to both sham and vehicle-treated groups. No significant differences were found between the respective control groups.

**Table 1:** Effect of PIs on *ex vivo* heart function parameters at baseline (n = 8).

	Sham	Vehicle	PI
**+dP/dt (mmHg/sec)**	1411.0±126.9	1068.8±70.9	633.1±57.7 *** **^, #^**
**−dP/dt (mmHg/sec)**	−966.8±71.9	−782.1±118.2	−338.4±31.0 *** **^, ##^**
**Heart rate (bpm)**	274.1±10.6	283.5±9.2	197.4±36.0

*Abbreviations: PI – protease inhibitor.*

*** *p<0.001 vs. sham; #p<0.05 and ##p<0.01 vs. vehicle.*

Data presented as mean ± SEM and two-way ANOVA with Bonferroni post-hoc test performed for statistics. No significant differences were found between the sham and vehicle groups, and statistical differences for PI are indicated with * or # symbols.

**Figure 3:**
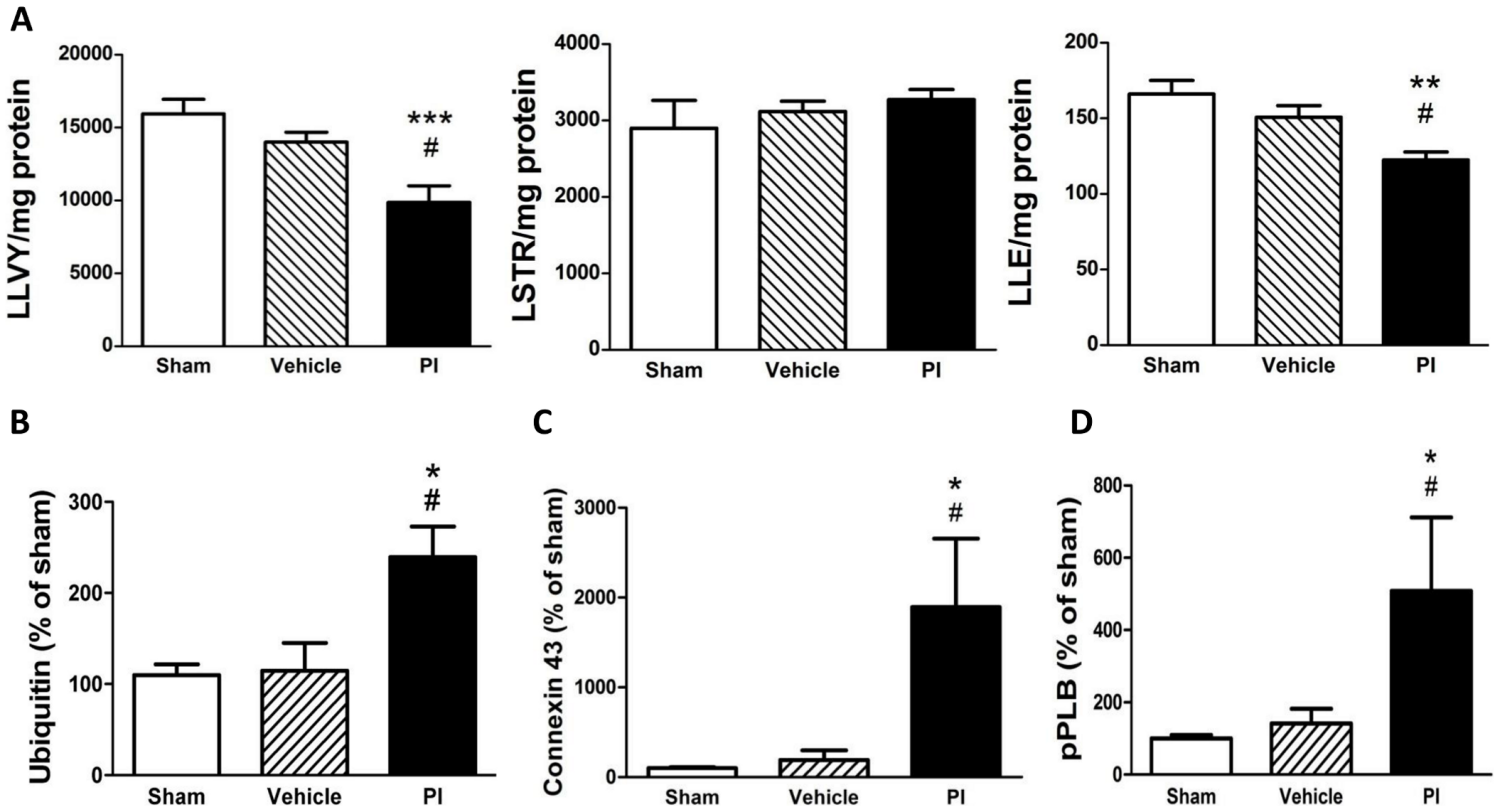
Proteasome and protein expression of contractile regulators following PI treatment (n = 8). A) LLVY (chymotrypsin-like activity), LSTR (trypsin-like activity), and LLE (caspase-like activity) of the proteasome; B) Total protein ubiquitination; C) Connexin 43; and D) Phosphorylated PLB. *p<0.05, **p<0.01, ***p<0.001 vs. sham; #p<0.05 vs. vehicle. Data presented as mean ± SEM and one-way ANOVA with Bonferroni *post-hoc* test performed for statistics. *pPLB – phosphorylated phospholamban, PI – protease inhibitor.*

We next evaluated the effects of PI treatment on the cardiac UPS system and our data demonstrate significantly lowered chymotrypsin-like and caspase-like, but not trypsin-like proteasomal activities (**Fig. 3A**). In parallel, global ubiquitination of total proteins significantly increased with PI administration (**Fig. 3B**). Since our earlier work pointed towards an alteration in SERCA-2a levels [17], we attempted to gain additional insight regarding PI-mediated contractile dysfunction, markers regulating this ion channel as well as a marker for electrical conductance. Here myocardial expression of the gap junction protein connexin 43 (marker for electrical conductance) and pPLB (SERCA-2a regulator) increased with PI treatment (**Fig. 3C, D**).

**Figure 4:**
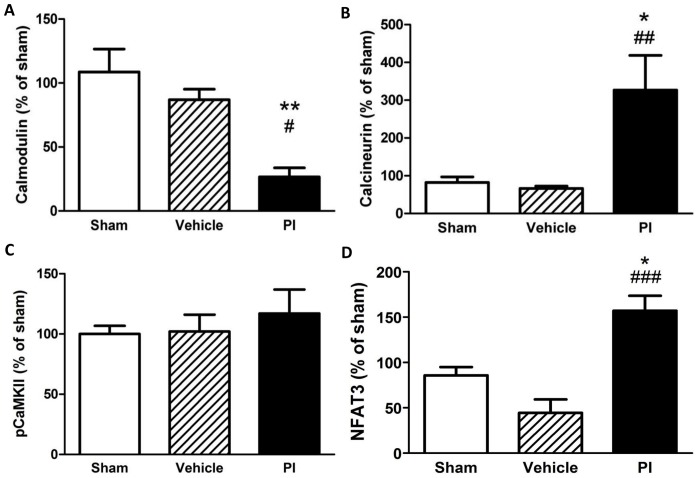
Calcium pathway protein expression in response to chronic PI therapy (n = 6–8). A) Calmodulin; B) Calcineurin; C) pCaMKII; and D) NFAT3. *p<0.05, **p<0.01 vs. sham; #p<0.05, ##p<0.01 vs. vehicle, ###p<0.001 vs. vehicle. Data presented as mean ± SEM and one-way ANOVA with Bonferroni *post-hoc* test performed for statistics. *NFAT3 – nuclear factor of activated T-cells 3, pCaMKII – phosphorylated CaMKII, PI – protease inhibitor.*

**Figure 5:**
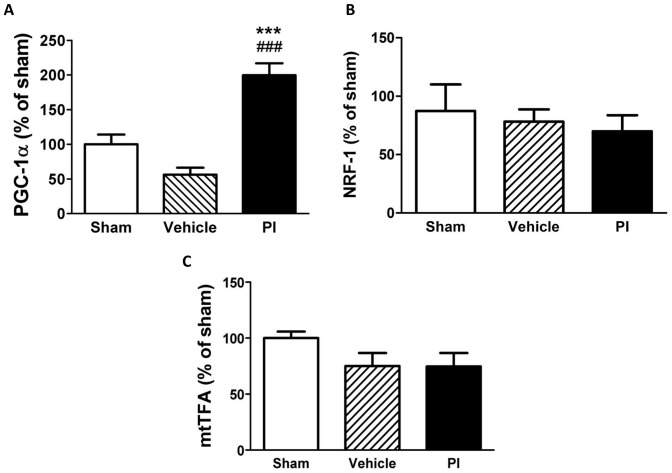
Myocardial peptide levels of transcriptional regulators of cardiac hypertrophy and mitochondrial biogenesis with 8 weeks PI therapy (n = 6–8). A) PGC-1α; B) NRF-1; and C) mtTFA. ***p<0.001 vs. sham; ###p<0.001 vs. vehicle. Data presented as mean ± SEM and one-way ANOVA with Bonferroni *post-hoc* test performed for statistics. *NRF-1 nuclear respiratory factor 1, mtTFA – mitochondrial transcription factor A, PGC-1α – peroxisome proliferator-activated receptor gamma coactivator-1-alpha, PI – protease inhibitor.*

We next assessed calcium and mitochondrial energetic signaling markers and found that PIs significantly downregulated the expression of the calcium-binding protein calmodulin (**Fig. 4A**), while calcineurin was upregulated (**Fig. 4B**). However, pCaMKII levels remained unchanged while nuclear NFAT3 expression increased versus controls (p<0.05 vs. sham and vehicle) (**Fig. 4**C, **D**). Here expression of PGC-1α was significantly upregulated in PI-treated heart tissue (**Fig. 5A**) while no changes were found for mitochondrial biogenesis markers (NRF-1, mtTFA) (**Fig. 5B, C**).

**Figure 6:**
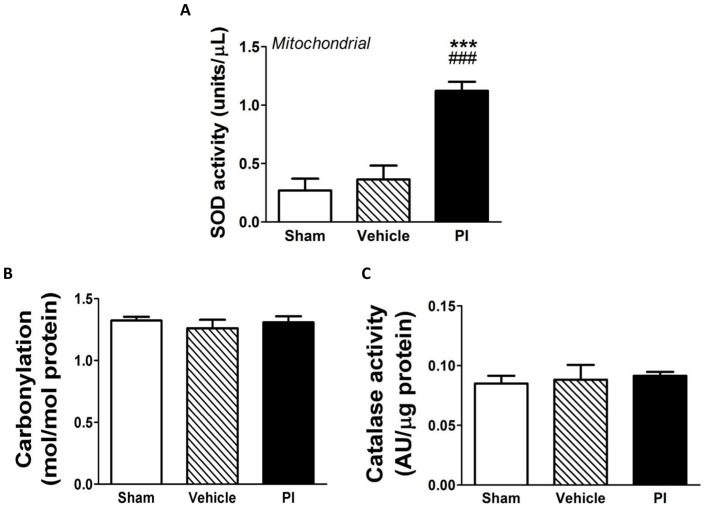
Myocardial oxidative stress profile (n = 8). A) Mitochondrial SOD activity; B) Protein carbonylation; and C) Myocardial catalase activity. ***p<0.001 vs. sham; ###p<0.001 vs. vehicle. Data presented as mean ± SEM and one-way ANOVA with Bonferroni *post-hoc* test performed for statistics. *PI – protease inhibitor, SOD – superoxide dismutase.*

**Figure 7:**
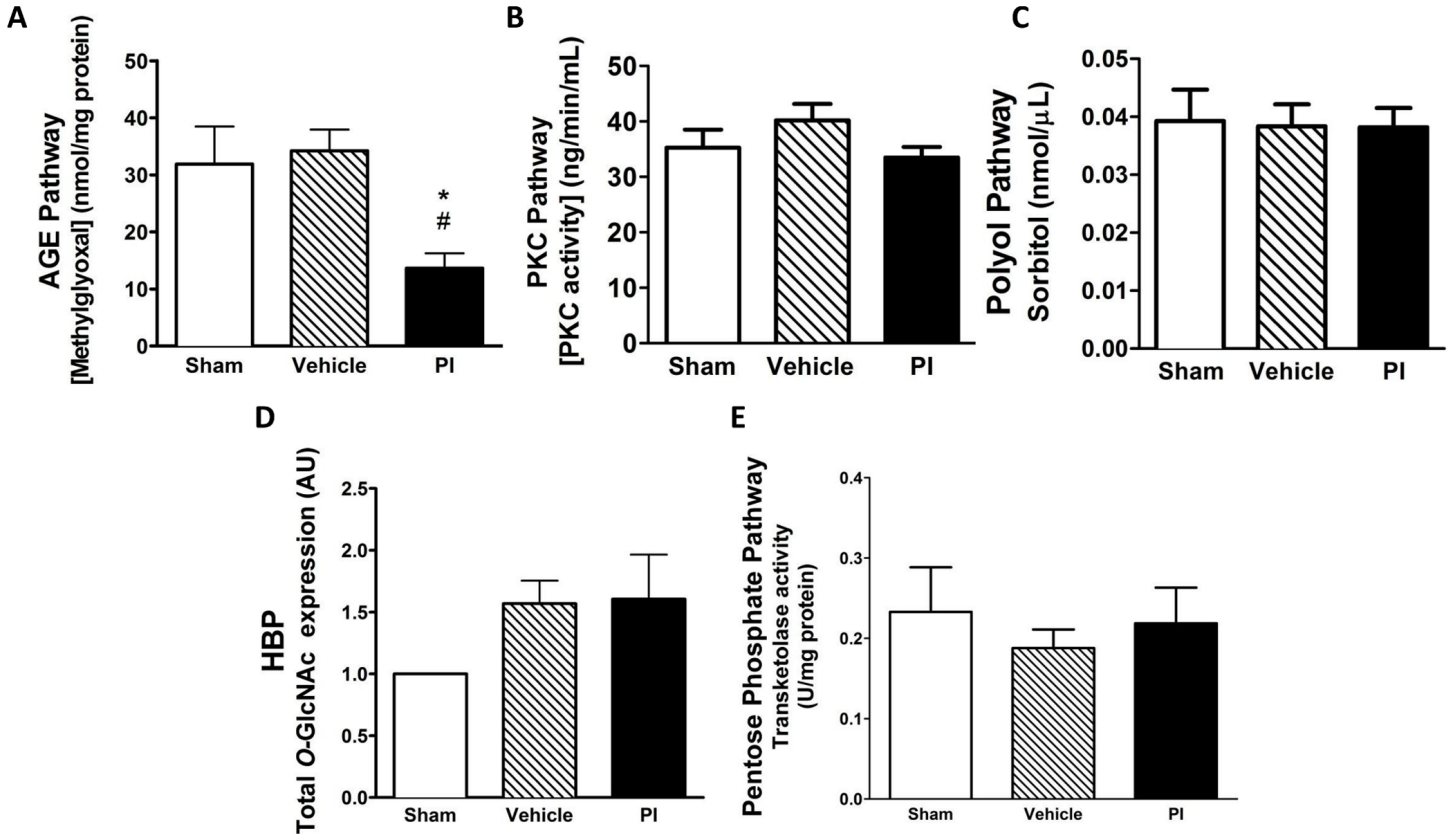
Profile of cardiac non-oxidative metabolic pathways (n = 8). A) AGE; B) PKC; C) Polyol pathway (D-sorbitol); D) HBP (*O-*GlcNAcylation); and E) Pentose phosphate pathway (transketolase). *p<0.05 vs. sham; #p<0.05 vs. vehicle. Data presented as mean ± SEM and one-way ANOVA with Bonferroni *post-hoc* test performed for statistics. *AGE – advanced glycation end-products, HBP – hexosamine biosynthetic pathway, PI – protease inhibitor, PKC – protein kinase C.*

Myocardial SOD activity within mitochondria was markedly upregulated (**Fig. 6A, B**) in PI-treated hearts. However, cardiac catalase activity and protein carbonylation did not differ for any of the experimental groups (**Fig. 6C, D**). The non-oxidative glucose pathways did not display any significant changes, except that PI treatment attenuated the AGE pathway, i.e. 6.25±0.52 vs. 14.87±2.05 nmol/mg protein (sham) (p<0.01) and vs. 13.16±2.23 nmol/mg protein (vehicle) (p<0.05) (**Fig. 7**).

## Discussion

Although HAART markedly improves the quality of life and prognosis of HIV-infected individuals, it also elicits cardio-metabolic side effects in the long-term. Since molecular mechanisms underlying this process are poorly understood, we evaluated early cardio-metabolic changes in a rat model of PI treatment. The main findings of this study are: 1) PI-treated rats exhibited lipid abnormalities; and 2) Rats exposed to PIs display altered myocardial ubiquitin proteasome and calcium-handling pathways together with decreased contractile function.

### 

#### PI-treated rats exhibited lipid abnormalities

Previous studies demonstrated that a significant proportion of HAART patients develops impaired glucose tolerance, IR and type 2 diabetes [11], [12]. Here our data revealed that PI-treated rats displayed elevated serum LDL-cholesterol and cardiac/hepatic tissue triglyceride levels, identifying perturbed lipid metabolism as a relatively early occurrence. Although not focusing on initial PI-mediated changes, previous work also reported that lipid derangements are one of the commonest side-effects triggered by Lopinavir/Ritonavir usage [42]. Moreover, clinical studies indicate that altered fat partitioning (i.e. lipodystrophy) is common with PI treatment [7], [43], [44] compared to overt increases in weight gain, and that this occurs within the first year of therapy. Our data indicate that the onset of IR could follow at a later stage in the progression of cardio-metabolic dysfunction following PI treatment. In support, the HOMA-IR assessment and several non-oxidative pathways of glucose metabolism (HBP, PKC, polyol pathway), that are usually strongly linked to IR and type 2 diabetes, were not activated in our model. However, the AGE pathway was unexpectedly downregulated and further studies are required to elucidate whether this is a direct effect or if it occurs as a result of other changes triggered by PI treatment.

How exactly does PI treatment induce the changes in lipid metabolism here observed? The mechanisms underlying higher food consumption with PI exposure are unclear, but well-known regulators of dietary intake (e.g. leptin, neuropeptide Y, ghrelin) may be implicated [45] and therefore form part of our ongoing investigations. PI treatment induced gene expression of *accβ* and *hmgcr* in the liver that would be expected to enhance fatty acid oxidation and cholesterol synthesis, respectively. There were also early signs of elevated cardiac *gpam* expression (although not statistically significant versus all matched controls), while it was robustly upregulated in adipose tissue (data not shown). The gene expression results therefore indicate that the higher serum LDL-cholesterol levels may result from greater adipose triglyceride synthesis and subsequent export to the liver and heart. Here increased hepatic *hmgcr* expression may enhance VLDL production and with a corresponding elevation in the availability of circulating LDL-cholesterol.

Since PIs may also have direct transcriptional effects that trigger gene expression, we also assessed whether SREBPs – well-known transcriptional regulators of several lipid and cholesterol synthesis genes [23] – are implicated in the observed gene induction. We found no significant differences when analyzing SREBP expression (gene and protein levels) in liver and heart tissues, and suggest that other transcriptional modulators that regulate lipid and cholesterol genes may be involved. An alternate explanation may relate to the fact that Ritonavir is a reversible and competitive inhibitor of specific 20S proteasome subunits [25]. Since the UPS also plays a key role to regulate SREBP-1 binding to target gene promoters (mediating its degradation) [27], [28], lower UPS activity may lead to more SREBP-1 remaining bound to gene promoter(s). This in turn could result in greater induction of target genes, even though total SREBP expression levels were unaltered. These possibilities are currently being pursued in our laboratory.

Together our study shows that early changes induced by PI treatment resemble the metabolic syndrome, a combination of risk factors that predispose to the future onset of IR, type 2 diabetes and CVD. Moreover, the higher serum LDL-cholesterol levels mirror a pre-atherogenic state that may eventually trigger the onset of various cardiac complications, e.g. acute myocardial infarction.

### Rats exposed to PIs display altered myocardial ubiquitin proteasome and calcium-handling pathways together with decreased contractile function

What are the underlying mechanisms whereby PI administration impairs contractile function? Our results show no significant remodeling of hearts exposed to PIs, i.e. lack of ultrastructural changes, fibrosis and cardiac hypertrophic response. We also evaluated markers for myocardial oxidative stress since others found a link between PI exposure and elevated ROS production [18]–[20], but found no evidence of damaging effects of myocardial oxidative stress at baseline (no changes in degree of protein carbonylation). However, PI-treated hearts exhibited augmented myocardial SOD activity suggesting that increased oxidative stress is blunted by intracellular defense systems. Thus, these data indicate that harmful effects of previously reported PI-induced ROS occur at a later stage during the HAART regimen. In agreement, there was no ROS-mediated induction of several non-oxidative glucose metabolic pathways in PI-treated rats. This contrasts our recent work where greater myocardial oxidative stress, HBP activation and apoptosis contributed to contractile dysfunction [35].

The heart functional data are consistent with our earlier work [17] and reveal attenuated contractile function without significant alterations to heart rate. Here the ±dP/dt findings implicate the myocardial calcium handling pathway, as diastolic calcium is a key determinant of contractile function and calcium signaling [46]. Since PI treatment decreased and increased myocardial UPS activity and ubiquitination, respectively, this may lead to an accumulation of contractile protein aggregates and impaired cardiac contractility and signaling pathways. For example, protein turnover of connexin 43, PLB and SERCA-2a are all regulated by the UPS [47]–[51] and may explain the higher expression levels found here and before by us [17]. Others have established that altered connexin 43 expression can precede arrhythmias, ventricular fibrillation and incorrect signal propagation in the long-term [52]–[57]. Therefore we tentatively suggest that elevated connexin 43 expression in our model may results in detrimental effects on contractile function in the future, especially within the context of HIV-AIDS.

We previously identified lower myocardial calcium levels and higher SERCA-2a protein expression with PI treatment [17], and now report attenuated and elevated calmodulin and pPLB expression levels, respectively. In parallel, we found increased myocardial calcineurin and NFAT3 expression levels. Of note, others found that cardiac-specific calcineurin overexpression resulted in enhanced pPLB and SERCA-2a expression and diminished phosphorylation and redistribution of connexin 43 [58]. This was associated with depressed contractility and cardiac hypertrophy. Here the authors proposed that connexin 43 may be a downstream target of calcineurin and that attenuated connexin 43 levels may be linked to perturbed gap junction assembly and arrhythmogenesis [58]. We propose that a similar scenario may exist in our model and that greater calcineurin activation is linked to elevated connexin 43 expression that may compromise gap junction function. Increased SERCA-2a, connexin 43 and pPLB expression may occur as a result of lower myocardial UPS and have also been implicated as downstream transcriptional targets of calcineurin [58]. Thus, elevated connexin 43 and pPLB expression may represent an adaptive response by PI-treated hearts to improve calcium handling, which may improve cardiac function. Higher calcineurin activation also leads to increased dephosphorylation and translocation of NFAT3 to the nucleus for activation of downstream targets, e.g. PGC-1α and pro-hypertrophic genes [59], [60]. However, since the calcineurin-NFAT3 pathway did not result in cardiac hypertrophy in our model, we are of the opinion that longer-term activation may eventually result in a hypertrophic response. These findings, however, represent a model of altered cardiac physiology and suggest a potential association with PI-induced molecular alterations to key junction and ionic proteins that may precede the onset of contractile dysfunction. Moreover, the metabolic side-effects elicited by PI treatment in our model – although at a relatively early stage – may also affect heart function as a downstream target. Thus we do not imply that the protein expression alterations are directly associated with the altered contractility found in our model. Data linking these phenomena are scarce and therefore makes definitive conclusions difficult. Together these findings indicate that perturbed calcium handling may contribute to the PI-mediated contractile dysfunction found in our experimental model in the longer term. However, further studies are required to confirm whether this is indeed the case.

Since myocardial PGC-1α was upregulated, this implies that PIs exert initial effects at the mitochondrial level. PGC-1α is a well-described transcriptional regulator of mitochondrial biogenesis [61], [62] and we propose that higher expression levels may represent an early compensatory response to energetic stress. In agreement with this notion, NRF-1 and mtTFA expression remained unaltered while we previously identified no changes for myocardial ATP levels and AMPKα expression following 8 weeks of PI administration [17]. It is likely that reduced UPS activity in PI-treated hearts may contribute to the increased PGC-1α levels here observed. In support, others established that lower UPS-mediated protein turnover in fibroblasts resulted in PGC-1α stabilization and mitochondrial biogenesis [63], while it can also be rapidly degraded in the nucleus [64]. The reason(s) as to why NRF-1 and mtTFA were not upregulated in response to PGC-1α remains unclear but could be a unique phenomenon within this animal model. Therefore we cautiously interpret our findings and conclude a potential association between proteasomal inhibition via PIs and activation of the PGC-1α pathway.

### Study limitations

The data here generated do not allow us to make a direct causal link between metabolic/molecular alterations and decreased heart function with PI treatment. We propose that additional studies that investigate more markers of electrical conductance, ion homeostasis and mitochondrial biogenesis would be useful to help answer these questions. Further, transgenic mouse studies to generate gene knockout/knockdown of molecular targets here identified, together with PI exposure should further advance our understanding of PI-mediated cardio-metabolic complications.

In conclusion, our study demonstrates that early changes triggered by PI treatment include increased serum LDL-cholesterol and myocardial triglyceride levels, together with decreased cardiac function. Furthermore, PI exposure inhibits the myocardial UPS and leads to elevated calcineurin and connexin 43 expression that may contribute to cardiac contractile dysfunction in the long-term. Our findings also highlights potential molecular targets that may have detrimental metabolic and contractile effects. Thus our study alerts to the association between PI treatment and cardio-metabolic side effects and we propose that further clinical studies are needed to evaluate these pathways in HIV+ patients on chronic HAART.
