# Correction: Cardio-Metabolic Effects of HIV Protease Inhibitors (Lopinavir/Ritonavir)

**DOI:** 10.1371/annotation/a1e2a42f-7f10-4654-a042-57d19de50208

**Published:** 2013-11-26

**Authors:** Kathleen M. S. E. Reyskens, Tarryn-Lee Fisher, Jonathan C. Schisler, Wendi G. O'Connor, Arlin B. Rogers, Monte S. Willis, Cynthia Planesse, Florence Boyer, Philippe Rondeau, Emmanuel Bourdon, M. Faadiel Essop

This article was republished on November 25, 2013, to replace an incorrectly published version. Please download this article again to view the correct version.

